# Texture-modified diets in long-term care facilities: assessing adherence to nutrition standards

**DOI:** 10.1016/j.jarlif.2026.100072

**Published:** 2026-05-22

**Authors:** Erica Deasy, Hannah Sheedy, Cathal O’ Hara, Niamh Walsh, Pauline Dunne, Maria McKenna, Marie Hannon, Maria Gleeson-Cary, Gráinne Kent

**Affiliations:** a School of Food and Nutritional Sciences, University College Cork, Ireland; b School of Population Health, Royal College of Surgeons in Ireland, Dublin, Ireland; c Nutrition and Dietetics Department, HSE Dublin and Midlands, Dublin, Ireland; d Nutrition and Dietetics Department, Our Lady’s Hospice & Care Services, Harold’s Cross, Dublin, Ireland; e Speech and Language Therapy Department, Brothers of Charity Services, Southern Region, Ireland

**Keywords:** Long-term care, Older adults, Modified texture diets, Dysphagia, Menu quality

## Abstract

**Introduction and aim:**

Texture-modified diets (TMDs) are commonly required for older adults but are associated with nutritional risks. The nutritional quality of TMDs provided in long-term care (LTC) facilities in Ireland is understudied. This study evaluated the nutritional quality of TMDs in one LTC facility against international menu standards and dietary reference values.

**Methods:**

A cross-sectional nutritional analysis of TMDs (International Dysphagia Diet Standardisation Initiative Levels 3–6) was conducted over two non-consecutive days in a residential long-term care facility in Ireland, encompassing all foods, drinks, and recipes as served over two typical days for Levels 3–6. Items were quantified and analysed using Nutritics (v6) and R (v4.3.3). Mean (SD) daily and per-meal energy and protein provision were compared with standards from the British Dietetic Association (BDA) Nutrition and Hydration Digest, the BDA Care Home Digest, and Canadian Menu Planning in LTC guidance. Other macro- and micronutrients were assessed against dietary reference values.

**Results:**

Mean daily energy and protein provision were below recommended standards for all TMDs, except energy at Level 3. Per-meal provision was also suboptimal for most meals, except protein at Level 3 for lunch. All TMDs exceeded recommended total and saturated fat ranges and failed to meet dietary fibre targets, while sodium met guidelines. All TMD Levels were below recommendations for vitamin D (except for Level 3), vitamin C, potassium, magnesium, iron and zinc.

**Conclusion:**

Routine TMD provision in this facility did not meet key nutrient standards, highlighting potential nutritional vulnerability among LTC residents. Improved menu planning, enrichment strategies, staff training, and clearer guidance are required, although further work examining intake and outcomes is needed.

## Highlights

• Texture-modified menus often failed to meet key energy and protein targets.
• Fibre, vitamin C, iron among nutrients consistently below reference values.
• Level 3 diets were higher in energy/protein but required excessive food volume.
• Vitamin D, E, K provision was frequently inadequate across levels.
• Improved texture-modified menu planning in Irish long-term care settings is needed.

## Graphical abstract

**Figure:**
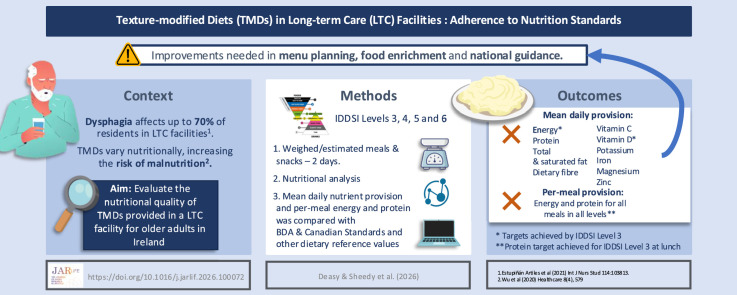

## 1 Introduction

Dysphagia, or difficulty swallowing, a common condition in older adults, affects up to 70% of residents of long-term care (LTC) facilities [1,2]. Although multifactorial, risk factors for dysphagia include dental status, neurological conditions such as stroke, progressive conditions, such as dementia and Parkinson’s disease, obstructive conditions, such as oesophageal stricture, and muscular issues, such as sarcopenia [[3], [4], [5], [6], [7]]. Often underdiagnosed and untreated in the older population, dysphagia can have a significant impact on an individual’s nutrient intake and is associated with an increased risk of malnutrition, poor quality of life and mortality [1,2,8]. Appropriate nutritional provision, through a texture-modified diet (TMD), required to ensure safe nutrition, is essential for individuals with dysphagia. However, TMDs are vulnerable to energy and nutrient losses due to the additional food processing required, e.g., chopping, tenderising, mincing, mashing and blending with liquid [[9], [10], [11]], thus increasing the risk of macronutrient and micronutrient inadequacy in meals provided to those with dysphagia [12].

The International Dysphagia Diet Standardisation Initiative (IDDSI) provides a framework of standardised terminology for food and beverage textures and thicknesses which aids the consistent implementation of TMDs for those with dysphagia [13]. The framework consists of 8 tiers ranging from Level 0 to Level 7, where Levels 0–4 describe beverage consistencies, i.e., thin, slightly thick, mildly thick, moderately thick and extremely thick, and Levels 3–7 describe food textures, i.e., liquidised, puréed, minced and moist, soft and bite-sized, easy to chew/regular. Despite the recognised importance of TMDs for individuals with dysphagia and the creation of standardised terminology through the IDDSI framework, there remains a lack of national guidelines for nutritional provision and monitoring in LTC facilities in Ireland [14]. This gap leaves texture-modified menus vulnerable to inconsistencies in nutritional content. While Ireland has food-based dietary guidelines for the general older population [15] and policies exist addressing nutrition for adults in acute hospitals and disability services [16,17], no equivalent yet exists for LTC settings for older adults, where the need for TMDs is high.

In contrast, the British Dietetic Association (BDA) in the UK provide food service nutrition standards for nutritionally vulnerable populations (e.g., those with dysphagia), namely the BDA Nutrition and Hydration Digest [18] and Care Home Digest [19]. Additionally, Dietitians of Canada, L’Ordre professionnel des diététistes du Québec (OPDQ) and the Canadian Malnutrition Task Force (CMTF) provide specific guidance on menu planning in LTC [20].

With approximately 32,000 beds in LTC facilities for older adults in Ireland [21] and an estimated 3.6 million similar bed types across Europe [22], plus a high prevalence of swallowing difficulties among residents, the provision of nutritionally appropriate TMDs represents a significant component of care, particularly in the context of population ageing and rising demand for LTC services [22,23]. Despite the widespread use of TMDs in LTC facilities, there are few studies internationally that have assessed the nutritional provision of TMDs in LTC against age-specific dietary reference values. In a systematic review of TMDs, puréed menus in three studies in LTC facilities from Denmark, the US and Canada respectively, were less likely to meet nutrient recommendations for dietary fibre, vitamin B6, folate, vitamins D, E, K, calcium, potassium, magnesium and zinc [12]. However, findings across these studies vary, reflecting differences in study design (single- versus multi-site), TMD definitions (e.g. blended or puréed) and in the nutrients assessed, which limits generalisability. Furthermore, to the author’s knowledge, no published Irish data has yet assessed nutritional provision of TMDs in LTC facilities against age-specific standards. The limited and heterogeneous nature of the available evidence highlights the need for IDDSI-level-specific nutrient analysis, including detailed macro- and micronutrient comparison against appropriate standards.

The aim of this study was to evaluate the nutritional quality of TMDs provided to residents in a LTC facility for older adults in Ireland, an understudied cohort, by assessing adherence to established UK and Canadian menu standards. Specifically, this novel study sought to assess the daily and per-meal nutrient content of the texture-modified menu compared with available recommended nutrient targets for energy, protein, total fat, dietary fibre and sodium. The provision of additional nutrients of concern relevant to individuals living in LTC was compared with relevant dietary reference values.

This study aims to evaluate TMD provision (menu quality), rather than actual dietary intake or clinical outcomes. Examining the nutrition provided to vulnerable older adults with dysphagia in LTC facilities represents an important first step in mapping the current nutritional landscape in LTC.

## 2 Methods

### 2.1 Study design and setting

This study employed a cross-sectional design to analyse texture-modified diets (TMDs) served on two non-consecutive, typical (non-holiday) days within a four-week cook-serve menu cycle in a long-term care (LTC) facility for older adults. Study days were selected using a pragmatic purposive sampling approach to capture typical routine service, within routine operational constraints. The study evaluated the nutritional provision of TMDs (IDDSI levels 3, 4, 5 and 6, as prepared and classified by the facility’s standard menu), representing the levels which involve the greatest degree of texture modification, against the available standards in the UK and Canada for food providers in LTC facilities [[18], [19], [20]] and other dietary reference values. The study was conducted in an urban LTC facility in Ireland, which at the time of the study (January/February 2024), accommodated 128 residents, 45% of whom required TMDs due to dysphagia. Ethical approval was obtained from the University College Cork Social Research Ethics Committee (2023–300).

### 2.2 Data collection

Data were collected by nutrition researchers (ED and HS) over three onsite visits to the LTC facility (from 19th January to the 2nd February 2024), with each researcher responsible for two IDDSI levels. Foods and drinks were collected as served, as standard portions prepared by catering staff at the point of service, reflecting usual portioning practice, with one serving collected for each food and drink offered at each applicable IDDSI level. Data encompassed two full days of provision for residents with dysphagia including breakfast, lunch, evening meal, starters/soups, desserts and snacks (including tea/coffee) across IDDSI levels 3–6. IDDSI levels were based on facility classification and were not independently verified. Beverages thickened in the kitchen (e.g., soups, smoothies) were collected as prepared prior to service, while drinks thickened at ward level (e.g., tea/coffee) were sampled prior to the addition of thickening agents. A hierarchical approach to quantification was adopted: food and beverages were weighed wherever possible and only when weighing was not feasible were alternative methods such as photographic food atlas [24], or household measures such as tablespoon, teaspoon, pint used. The individual components of each meal, e.g., meat, potato, vegetables etc., were weighed using two identical calibrated, portable digital food scales (Tanita, Japan) to ensure consistency. Composite meals, e.g., Shepherd’s pie, were weighed whole. Individual recipe ingredients, at brand level where possible, excluding commercial thickeners, and detailed information on preparation methods for texture-modified meals was obtained from the TMD chef (trained onsite by an external practitioner with expertise in IDDSI implementation) and the catering manager. The raw data set included meal portions (g), individual meal ingredients (g), recipes and preparation methods (e.g., boiled, fried).

### 2.3 Data entry, analysis and evaluation

Nutrient composition of the meals were estimated using UK and Irish food composition databases via Nutritics© version 6 [25]. Researchers engaged in comprehensive Nutritics© training to ensure competency and confidence in data entry. Each researcher entered data for their assigned IDDSI level. Individual foods and drinks were added directly and composite meals were added via recipes created using their ingredient lists, quantities and cooking methods. Commercial thickeners were not included in the nutritional analysis. All entries were cross-checked for accuracy, including verification of shared ingredients across common recipes. Any discrepancies were reviewed by a third researcher (GK) and consensus was reached among all three researchers. The resulting nutritional content of the meals was exported to Microsoft Excel, cleaned and then exported to R (version 4.3.3) for statistical analysis [26]. The mean and standard deviation (SD) daily provision of nutrients were calculated for each IDDSI level (hereafter described as Level 3, 4, 5 & 6) for energy (kcal), protein (g), total fat (g), saturated fat (g), total carbohydrate (g), free sugars (g), dietary fibre (g), riboflavin (mg), total folate (µg), vitamin B12 (µg), vitamin C (mg), vitamins D, E, K (µg), sodium (mg), potassium (mg), calcium (mg), iron (mg), magnesium (mg) and zinc (mg) and per-meal nutrient content for energy (kcal) and protein (g). These nutrients were chosen as they were identified as nutrients of concern for older adults or were already shown to be low in LTC menus [15,20].

The mean daily energy and protein content of the TMDs provided at the LTC facility were compared with available international guidance for the population, including the BDA Nutrition and Hydration Digest, the BDA Care Home Digest and the Canadian Menu Planning in Long-Term Care guidance. Mean daily total fat (percentage of energy), dietary fibre and sodium content were assessed against targets specified in the Canadian guidance. At meal level, the mean energy and protein content of individual texture-modified meals were compared with the BDA Nutrition and Hydration Digest targets for meals and snacks for nutritionally vulnerable adults. Mean protein provision across meals was further compared with the BDA Care Home Digest and the Canadian Menu Planning in Long-Term Care guidance recommendations for protein distribution across the day (Appendix 1).

In the absence of LTC-specific daily standards for other nutrients, provision of additional nutrients of concern, including saturated fat, carbohydrates and selected micronutrients, was analysed and compared with dietary reference values set by the Scientific Advisory Committee on Nutrition (SACN), the European Food Safety Authority (EFSA) and the Institute of Medicine (IOM) if values were not set by SACN/EFSA. The vitamin D recommendation of 20 µg was used in line with guidance from the Food Safety Authority of Ireland (FSAI) guidance specifically for older adults who are housebound (Appendix 1).

## 3 Results

### 3.1 Mean nutrient provision relative to menu standards

Table 1 outlines the total and per-meal mean (SD) nutrient provision from texture-modified diets (TMDs) provided in a long-term care (LTC) facility. Compared with the daily nutrient targets recommended by the BDA Nutrition and Hydration Digest (1840–2772 kcal; 79–92 g protein), BDA Care Home Digest (2000 kcal; 75 g protein) and the Canadian Menu Planning in Long-Term Care guidance (≥2000 kcal; 100 g protein), mean daily energy and protein provision for all TMD Levels were below recommendations, with the exception of energy at Level 3. Across the two days of testing, total mean daily energy provision ranged from 1321 to 1992 kcal, with only Level 3 meeting the lower end of the recommended energy targets. Mean daily protein provision ranged from 45 to 74 g protein, thereby not meeting daily protein targets. All levels exceeded the daily total fat target recommended by the Canadian Menu Planning in Long-Term Care guidance (30–35% of energy from fat), ranging from 36 to 41% of energy (Level 3: 41% energy; Level 4: 36% energy; Level 5: 36% energy; Level 6: 38% energy). None of the levels met the daily dietary fibre target of 30 g recommended by the Canadian Menu Planning in Long-Term Care guidance, with mean daily dietary fibre provision ranging from 13 to 19 g. All levels met the sodium target of <3500 mg/day (Canadian Menu Planning in Long-Term Care guidance), with mean daily sodium provision ranging from 917 to 1516 mg.

**Table 1:** Total and per-meal nutrient provision from texture-modified meals provided in the long-term care facility.

	Breakfast	Lunch				Evening Meal				Snacks & Drinks				Total			
IDDSI Level	3–6*	3	4	5	6	3	4	5	6	3	4	5	6	3	4	5	6
Mean (SD)																	
Energy (kcal)	397 (0)	770 (58)	561 (33)	398 (17)	353 (13)	534 (99)	436 (0)	305 (39)	379 (52)	291 (178)	163 (0)	221 (35)	377 (35)	**1992** (334)	1557 (34)	1321 (91)	1506 (75)
Protein (g)	10.1 (0)	**32.7** (6.9)	19.2 (0.4)	14.2 (1.1)	13.5 (0.3)	20.5 (2.8)	16.4 (0.8)	8.9 (0.4)	17.7 (5.7)	10.9 (4.1)	7.1 (0.0)	11.3 (1.3)	11.4 (1.3)	74.1 (0.0)	52.8 (1.2)	44.5 (2.8)	52.8 (4.6)
Total fat (g)	10.5 (0)	40.0 (1.7)	26.0 (1.3)	19.8 (2.1)	17.7 (1.1)	27.5 (2.9)	18.4 (2.2)	14.2 (0.8)	23.2 (1.2)	13.7 (7.4)	7.7 (0.0)	7.9 (2.5)	12.8 (2.5)	91.6 (12)	62.5 (1.0)	52.4 (0.4)	64.2 (2.6)
Saturated fat (g)	5.0 (0)	23.1 (0.2)	13.0 (0.4)	11.1 (1.1)	10.0 (0.9)	15.8 (0.1)	7.8 (1.1)	8.5 (0.3)	11.2 (1.1)	7.9 (2.9)	5.3 (0.0)	5.1 (1.5)	4.5 (1.5)	51.8 (2.8)	31.1 (0.8)	29.7 (0.1)	30.7 (0.5)
Carbohydrate (g)	65.9 (0)	69.6 (17.3)	62.6 (5.0)	40.8 (9.9)	34.8 (0.8)	51.1 (15.5)	51.2 (4.2)	35.3 (12.2)	25.0 (4.7)	31.0 (23.7)	16.1 (0.0)	26.2 (4.7)	54.1 (4.7)	217.5 (56.5)	**195.8** (9.2)	**168.2** (26.9)	**179.7** (8.7)
Free sugars (g)	0 (0)	11.3 (0.8)	13.0 (1.8)	13.3 (0.8)	10.8 (0.0)	11.0 (1.0)	13.9 (0.4)	12.6 (2.4)	10.4 (0.1)	6.8 (7.0)	3.6 (0.0)	1.8 (0.0)	13.5 (0.0)	**29.1** (8.8)	**30.5** (2.2)	**27.7** (3.2)	**34.7** (0.2)
Dietary fibre (g)	6.3 (0)	7.2 (2.4)	8.0 (0.1)	4.6 (1.3)	4.3 (2.1)	1.3 (0.2)	4.6 (0.3)	1.9 (1.2)	1.1 (0.2)	0.4 (0.6)	0.1 (0.0)	0.3 (0.4)	1.8 (0.4)	15.2 (3.2)	19.0 (0.4)	13.1 (2.1)	13.5 (2.8)
Riboflavin (mg)	0.4 (0)	1.2 (0.1)	0.3 (0.0)	0.4 (0.0)	0.4 (0.0)	1.2 (0.2)	0.3 (0.1)	0.4 (0.0)	0.6 (0.3)	0.7 (0.2)	0.5 (0.0)	0.5 (0.0)	0.4 (0.0)	**3.5** (0.5)	1.5 (0.1)	**1.7** (0.1)	**1.7** (0.3)
Total folate (µg)	99.2 (0)	381.4 (53.9)	120.1 (1.1)	116.9 (21.6)	113.5 (37.9)	337.0 (50.7)	101.6 (2.1)	104.8 (4.2)	108.0 (38.3)	175.2 (53.9)	117.1 (0.0)	130.2 (0.0)	97.3 (0.0)	992.8 (158.5)	438.0 (1.1)	451.0 (25.7)	418.0 (0.4)
Vitamin B12 (µg)	0.6 (0)	2.4 (0.3)	0.5 (0.0)	1.1 (0.2)	1.1 (0.2)	2.8 (0.3)	0.8 (0.4)	1.1 (0.4)	2.0 (1.2)	1.1 (0.4)	0.7 (0.0)	0.7 (0.0)	0.6 (0.0)	**6.7** (0.9)	2.5 (0.4)	3.5 (0.6)	**4.2** (1.4)
Vitamin C (mg)	0.2 (0)	31.7 (14.0)	35.4 (0.2)	26.5 (16.6)	27.2 (23.7)	4.0 (0.8)	19.2 (1.5)	4.0 (1.1)	2.1 (2.9)	0.6 (0.8)	0.0 (0.0)	0.0 (0.0)	9.0 (0.0)	36.4 (15.6)	54.7 (1.7)	30.7 (17.7)	38.5 (20.8)
Vitamin D (µg)	2.2 (0)	9.3 (0.8)	1.6 (0.0)	2.3 (0.2)	2.1 (0.1)	9.7 (1.4)	1.6 (0.1)	3.0 (0.3)	3.8 (2.8)	4.4 (1.4)	2.8 (0.0)	3.3 (0.0)	2.5 (0.0)	**25.6** (3.6)	8.2 (0.1)	10.8 (0.1)	10.6 (2.6)
Vitamin E (µg)	2.6 (0)	8.4 (0.4)	2.3 (0.0)	2.4 (0.5)	2.5 (0.4)	7.3 (0.9)	1.6 (0.0)	2.4 (0.1)	3.3 (1.2)	3.4 (1.1)	2.3 (0.0)	2.5 (0.0)	2.2 (0.0)	**21.6** (2.3)	8.7 (0.0)	9.9 (0.4)	**10.6** (0.8)
Vitamin K (µg)	0 (0)	64.8 (26.3)	68.6 (0.0)	37.8 (27.7)	44.1 (36.1)	0.5 (0)	34.0 (3.2)	0.8 (0.4)	2.8 (2.7)	0 (0)	0.0 (0.0)	1.5 (0.0)	1.6 (0.0)	65.3 (26.3)	**102.6** (3.2)	40.2 (28.0)	48.4 (38.9)
Sodium (mg)	78.1 (0)	786.6 (239.1)	383.8 (17.0)	543.8 (22.1)	508.6 (51.5)	425.0 (88.7)	323.4 (51.3)	241.7 (42.4)	381.0 (84.9)	226.6 (110.3)	131.6 (0.0)	202.5 (43.1)	256.5 (43.1)	**1516.3** (40.1)	**916.9** (34.3)	**1066.1** (22.8)	**1224.4** (76.5)
Potassium (mg)	694.6 (0)	761.1 (328.1)	891.1 (28.3)	643.0 (11.6)	541.8 (171.7)	275.9 (79.9)	667.6 (125.2)	257.8 (100.8)	297.7 (30.1)	309.1 (113.1)	230.2 (0.0)	147.1 (0.0)	490.3 (0.0)	2040.7 (521.1)	2483.5 (96.9)	1742.5 (112.4)	2024.1 (201.8)
Calcium (mg)	251.2 (0)	844.9 (95.9)	255.0 (8.5)	252.4 (26.7)	212.4 (55.4)	771.3 (128.3)	216 (2.7)	246.9 (19.6)	291.6 (86.2)	453.1 (147.1)	304.1 (0.0)	261.0 (0.0)	159.5 (0.0)	**2320.5** (371.2)	**1026.3** (11.2)	**1011.5** (46.2)	914.7 (141.6)
Iron (mg)	2.7 (0)	1.6 (0.9)	2.7 (0.0)	1.6 (0.3)	1.7 (0.0)	0.9 (0.3)	1.7 (0.4)	0.8 (0.2)	1.7 (1.6)	0.3 (0.4)	0.0 (0.0)	0.0 (0.0)	0.5 (0.0)	5.5 (1.6)	6.3 (0.3)	5.1 (0.5)	6.6 (1.6)
Magnesium (mg)	72.9 (0)	44.0 (21.9)	72.9 (0.0)	36.0 (3.5)	30.5 (9.5)	19.6 (7.1)	43.1 (5.1)	18.5 (7.8)	18.9 (6.2)	24.5 (10.3)	17.4 (0.0)	10.9 (0.0)	39.7 (0.0)	161.0 (39.3)	186.7 (2.5)	138.3 (11.4)	162.0 (3.3)
Zinc (mg)	1.2 (0)	1.0 (0.6)	1.2 (0.0)	1.6 (0.4)	1.6 (0.3)	0.5 (0.1)	1.5 (1.1)	0.5 (0.1)	1.5 (0.4)	0.7 (0.2)	0.6 (0.0)	0.0 (0.0)	0.3 (0.0)	3.4 (0.9)	4.2 (1.0)	3.4 (0.3)	4.6 (0.8)

Values are presented as the mean of two non-consecutive days. **Bold i**ndicates values meeting relevant dietary reference values or guideline targets (daily or per-meal, as applicable). Carbohydrate values were expressed as percentage of total energy intake for comparison with recommendations. Protein (Level 3) was assessed against per-meal targets (BDA Nutrition and Hydration Digest). Total folate is not directly comparable to dietary folate equivalents (DFE) *Values for breakfast were the same across Levels 3–6.

With the exception of protein provision at lunch for Level 3, per-meal mean energy and protein provision across all TMD Levels were below meal-level targets outlined in the BDA Nutrition and Hydration Digest (breakfast: 545 kcal, 18 g protein; lunch and evening meal: 800 kcal, 27 g protein; snacks and drinks: 564 kcal, 20 g protein), the BDA Care Home Digest (breakfast: 20 g protein; lunch 30 g protein; evening meal 25 g protein) and the Canadian guidance (breakfast: 20–25 g protein; lunch and evening meal: 25–30 g protein; snacks and drinks 25 g total). At breakfast, all levels consisted of the same meal and provided a mean (SD) of 397 (0) kcal and 10 (0) g protein; below recommended targets. Lunch showed greater variability, with mean energy provision ranging from 353 to 770 kcal and protein from 14 to 33 g. Level 3 provided the highest energy and protein, with protein meeting per-meal recommendations. Evening meals for all levels did not meet per-meal energy and protein targets, providing 305–534 kcal and 9–21 g protein. Snacks and drinks contributed limited energy and protein across all levels, ranging from 163 to 377 kcal and 7 to 11 g protein.

### 3.2 Mean nutrient provision relative to dietary reference values

Mean daily saturated fat provision exceeded the SACN recommended intake of <10% energy [27] for all TMD Levels, ranging from 18–23% of energy. For carbohydrates, EFSA recommends that 45–60% of total energy is derived from carbohydrate [28]. TMD Levels provided a range of 44–51% of energy from carbohydrates, with only Level 3 falling below the recommended range (44% of energy from carbohydrates). All levels exceeded the WHO and SACN recommendation that <5% of energy should come from free sugars but remained below the upper limit of 10%, contributing 6–9% of energy across levels.

Among B vitamins, mean daily provision of riboflavin ranged from 1.5–3.5 mg across levels, with only Level 4 (1.5 (0.1) mg) falling below the EFSA PRI of 1.6 mg/day. Mean daily total folate provision ranged from 418–993 µg across all levels (which is likely an underestimation of dietary folate equivalents [DFE] as it does not take the higher bioavailability of folic acid e.g., from fortified foods into consideration), exceeding the EFSA PRI of 330 µg/day DFE [29]. Mean daily vitamin B12 provision ranged from 2.5–6.7 µg, with Level 3 and Level 6 meeting the EFSA AI of 4 µg/day [30].

Mean daily vitamin C provision was below the EFSA PRI (95–110 mg/day) for all levels, ranging from 31–55 mg/day [31]. For vitamin D, only the Level 3 diet exceeded FSAI recommendation of 20 µg/day (housebound older adults), providing 26 (4) µg [15]. For vitamin E, the Level 3 diet exceeded EFSA AI (11 mg) at 22 (2) mg and met the recommendation at Level 6 (11 (1) mg), but Levels 4 and 5 were below the reference value [30]. For vitamin K, only the Level 4 diet exceeded the EFSA AI (70 µg/day), providing 103 (3) µg/day, while the remaining levels ranged from 40–65 µg/day [30].

Regarding minerals, mean daily calcium provision exceeded the EFSA PRI (950 mg/day) for all levels except Level 6 (914 (142) mg), with the highest contribution at Level 3 (2321 (371) mg), nearing the tolerable upper intake level (UL) of 2500 mg [29,30]. All levels were below the potassium EFSA AI of 3500 mg/day, with mean daily provision ranging from 1743–2484 mg [30]. Mean daily iron provision was below the EFSA PRI of 11 mg/day for all levels, ranging from 5–7 mg [30]. Mean daily magnesium provision for all levels ranged from 138–187 mg, falling below the EFSA AI (300 mg/day) [30]. Mean daily zinc provision also fell below the EFSA PRI of 7.5 mg/day across all levels, ranging from 3.4–4.6 mg [30].

## 4 Discussion

This study evaluated the nutritional quality of TMD provision (menu quality) in a LTC facility for older adults in Ireland, against established nutrition standards from the BDA Nutrition and Hydration Digest, the BDA Care Home Digest and the Canadian Menu Planning in Long-Term Care guidance. Additional nutrients of concern were compared to relevant dietary reference values. As such, this analysis examines provision, rather than residents’ dietary intake or clinical outcomes, representing an important first step in mapping the nutritional landscape for older adults with dysphagia in LTC facilities.

This study identified notable imbalances in the composition of the TMDs. Across levels, daily energy and protein provision failed to meet recommended targets, with the exception of energy at Level 3. At meal level, energy and protein provision were below per-meal standards across all meals and textures, with the exception of protein at lunch for Level 3. In contrast, all levels exceeded the recommended daily total and saturated fat targets, while none met the daily dietary fibre recommendation. Sodium provision, however, was within the recommended limit for all levels. Carbohydrate provision largely fell within the reference range, with the exception of Level 3, and free sugars remained below the recommended upper threshold.

Micronutrient provision was below dietary reference values for several nutrients. Across all levels, several nutrients of relevance to older adults, including vitamin C, potassium, iron, magnesium and zinc, were below dietary reference values. Fat-soluble vitamin provision varied across levels, with vitamin D meeting recommendations only at Level 3, vitamin E at Level 3 and 6 and vitamin K at Level 4. Among B-vitamins, riboflavin and vitamin B12 provision were below recommendations for Levels 4 and 4–5 respectively, whereas folate met recommended targets across all Levels. Calcium provision generally met recommended targets but approached the tolerable upper intake level at Level 3 and fell below recommendations at Level 6.

### 4.1 Comparison with menu standards

The findings of this study are consistent with other research reporting variable nutrition provision from TMDs [12,32] and lower energy and protein content compared to regular diet [10,33,34]. It is significant and concerning that most levels did not provide enough energy and protein given that those with dysphagia are at risk of malnutrition and sarcopenia, functional decline and frailty, all ultimately detrimentally impacting residents’ quality of life [[35], [36], [37], [38]]. This finding is likely the result of inadequate and/or inconsistent food-based enrichment strategies (also called food fortification) employed at kitchen level. These strategies use everyday ingredients (milk, cream, cheese etc.) to increase the nutritional content of diets; a well-documented and supported approach to improve nutritional intake in LTC facilities [27,28].

The Level 3 diet provided higher daily energy and lunch-time protein; a reflection of how Level 3 meals were prepared in the LTC facility. All Level 3 meals were liquidised using whole milk as the dilutant, increasing the energy and protein content of the meals and therefore the overall daily provision. While a form of food enrichment, this process also substantially increased the total volume of food required to be consumed. As a result, for residents with a significantly restrictive texture modification (Level 3), a much larger quantity of food must be consumed to meet their nutritional needs (Level 3: 2471 (268) g vs 1759–1839 g for Levels 4–6; data not shown). This is significant given that TMD consumers are often unable to consume a full meal (however, literature regarding meal consumption of individual IDDSI-specific levels is lacking) [12]. Therefore, while food-based enrichment strategies were implemented by catering staff, their execution may require further optimisation. The nutritional training needs of healthcare catering staff are poorly understood and targeted training may be required to support both IDDSI-compliant TMD provision and optimisation of nutritional quality [39].

Regarding per-meal nutrient targets, a significant finding was that snacks, including drinks, offered minimal nutritional content (163–377 kcal; 7–11 g protein). Snacks have the potential to contribute meaningfully to energy intake in residents in LTC facilities [40] and should be optimised for those with dysphagia to support nutritional provision. In practice, texture-modified snacks may be less accessible due to a limited range of suitable options, fewer routinely available offerings and reliance on staff or visitors for preparation and assistance, which may contribute to lower overall intake [41]. Safe and nutritionally appropriate snack options are needed in LTC settings, offering opportunity for commercialisation of ready-made texture-modified snacks.

Total fat provision from all the TMD Levels exceeded recommendations (30–35% energy), providing 36–41% of energy, reflecting a reliance on fat, e.g., butter, cream, whole milk to increase energy content and emphasising the importance of menu planning and catering training in nutrition support [19,20]. While the addition of these foods is an important enrichment strategy and can increase energy-density and protein-density in the case of milk, other nutrient-dense foods (e.g., nuts and nut butters, eggs, pulses, skimmed milk powder) can also be incorporated to enhance the overall nutritional quality of the menu.

Sodium content from all the TMD Levels was below recommended targets (917–1516 mg/day vs <3500 mg/day), however, despite how detailed the data collection process was, it is possible that not all discretionary salt added during the cooking process was recalled as part of the ingredients. There is little existing literature regarding the sodium content of TMDs [12] and further investigation of nutritional provision in TMDs, including sodium, is warranted .

Dietary fibre content was significantly lower than recommended targets. This finding is consistent with the literature reporting low fibre content of TMD menus, which is likely related to the avoidance of high-fibrous foods (e.g., wholegrains, husks, some fruits and vegetables) in TMDs [12,39]. Studies globally show that up to 80% of adults over 65 in LTC facilities are chronically constipated along with a high prevalence of laxative use in LTC facilities (55–67%) [42]. While there are a considerable number of causes of constipation in the older resident, some of which diet may not have an impact on, increasing fibre content of the diet is first-line dietary guidance [43].

### 4.2 Comparison with dietary reference values

The additional nutrients analysed and discussed in this paper were selected as they represent key nutrients of public health concern for this population and/or provision of these nutrients has already been shown to be low in LTC menus [15,20].

The daily provision of saturated fat in all the TMD Levels was double that of the SACN reference value, of <10% of energy (18–23% energy). This reflects reliance on the use of animal fat sources, such as whole milk, cream and butter to enrich the TMDs. In general, the daily provision of total carbohydrates and free sugars were within recommendations, with the exception of Level 3, which was slightly below (44% energy) the recommended reference range for carbohydrate (EFSA 45–60% energy), due to a higher provision of energy from fat.

Folate and related B-vitamins, including riboflavin and vitamin B12 have been linked to adverse health outcomes in older adults, including cardiovascular disease, cognitive dysfunction and osteoporosis [44]. While the causes of B-vitamin deficiency in this population are different for each nutrient, including malabsorption and adverse drug-nutrient interactions, deficiency may also be related to increased requirements for the nutrients and inadequate intakes [44]. In this study, the provision of B-vitamins assessed generally met or exceeded the dietary reference values, with the exception of riboflavin for Level 4 (1.5 (0.1) mg vs EFSA PRI 1.6 mg/day) and vitamin B12 for Level 4 and 5 (2.5 (0.4) µg and 3.5 (0.6) µg vs EFSA AI 4µg/day respectively). This is likely related to the use of vitamin-fortified brands by the catering department and emphasises the importance of fortified food products in supporting nutrient provision, especially given increased bioavailability of synthetic forms of B-vitamins [[44], [45], [46]]. However, data on dietary intake and nutrient status are needed to fully understand how these menu provisions translate into resident nutritional adequacy.

Vitamin C provision was low across all levels (31–55 mg vs EFSA PRI: 95–110 mg/day), a concerning finding given vitamin C’s roles in antioxidant defence, iron absorption, immune function and maintenance of skin integrity and wound healing (particularly relevant in older adults with a high prevalence of pressure ulcers) [47,48]. Current data on vitamin C intake and content of TMDs is limited, however given its importance for older adults, should be optimised [12].

Vitamin D is essential for older adults for bone health and its deficiency increases the risk of fractures. It is obtained via skin exposure to sunlight in the extended summer months (April to September), however, for older adults, particularly those dependent on LTC care, spending much of their time indoors, this is difficult to achieve [15,49,50]. While vitamin D can be obtained from foods, including natural sources (e.g., oily fish, eggs) and fortified foods (e.g., fortified ready-to-eat breakfast cereals, milk), generally supplementation is required to meet the higher FSAI recommendation for older adults with limited light exposure of 20µg/day [15,50]. In this study, the Level 3 diet met this recommendation, due to the use of vitamin-fortified milk as a dilutant, however, whether residents requiring this TMD consumed the entire volume of food provided is unknown. Vitamin E and K, other fat-soluble vitamins previously shown to be insufficient in LTC menus [10,51,52], were also suboptimal in this study, especially in Levels 4 and 5 for vitamin E and in Levels 3, 5 and 6 for vitamin K, potentially highlighting a lack of dietary variety, including plant-based oils and green leafy vegetables.

Regarding minerals, calcium provision met recommendations for most levels, with the exception of Level 6 (914 (142) mg). Notably, the Level 3 diet provided a calcium content (2321 (371) mg) approaching the EFSA upper level of 2500mg/day [29] (largely attributable to the substantial volume of milk incorporated in Level 3). This study did not assess supplementation use, however, Level 3 diet consumers who also take calcium supplements may be at risk of excess calcium intake. Data on dietary intake and status are needed to confirm this. In contrast, other minerals of concern; potassium, magnesium, iron and zinc, were all below dietary reference values for all levels, pointing to potential shortcomings in menu planning for a nutritionally vulnerable cohort.

### 4.3 Implications for practice and future directions

Importantly, this study assessed the nutritional provision of meals rather than consumption and therefore reflects menu-level provision within routine LTC practice. Concerning nutritional patterns were observed across the TMD Levels, including low energy and protein, very low dietary fibre, high saturated fat and low provision of key micronutrients, such as vitamins C and D, potassium, iron, magnesium and zinc. Addressing these issues requires coordinated action by LTC menu planning stakeholders, including catering management, dietitians, directors of nursing and LTC management, with engagement of resident representatives. Increasing daily food variety, particularly through greater inclusion of plant-based protein sources, legumes, fruit and vegetables, could improve fibre provision while reducing saturated fat ingredients. To address the low energy and protein content observed, food-based enrichment strategies should be expanded beyond the use of milk and cream to include a wider range of nutrient-dense ingredients. The use of fortified food products may also help address micronutrient gaps and improve overall nutrient-density. Although residents may request additional snacks or extra portions to boost meal provision, many residents depend on staff and/or visitors to recognise when intake is insufficent and provide partial (e.g., hand-over-hand) or full assistance [53]. The literature suggests that TMD consumers are less likely to complete all meals [12] highlighting the need for menus to cater to the higher end of nutritional provision. Those consuming TMDs are more likely to require greater clinical and nutrition support, including screening for and management of malnutrition risk, appropriate use of oral nutritional supplementation (ONS); micronutrient supplementation and laxatives, all of which necessitate general practitioner, dietetic and community pharmacist involvement. Given that polypharmacy is a significant problem for older adults [54], optimising the nutritional quality of TMD provision is in the interest of both resident and healthcare resource use. While the appropriate use of ONS is an essential component of nutrition support, improved nutrition, including food-based enrichment strategies can be effective, well-tolerated and cost-effective in improving dietary intake in older adults, offering benefits for the older adult, the care facilities and prescribing practices [55].

The focus on older adults in LTC, an often-underrepresented group in literature, provides new perspectives and highlights the need for integrated strategies to improve routine care practices for this vulnerable group. In Ireland, the absence of national guidance specific to food and hydration provision for older adults in LTC represents a significant gap. The Health Service Executive (HSE) Healthy Eating Active Living Implementation Plan outlines an intention to initiate the development of a Food and Hydration Policy for Older Persons Services in 2026, however, the timeline for full development and implementation remains uncertain [56]. The Irish National Standards for Residential Care Settings for Older People [57], produced by the Health Information and Quality Authority (Ireland’s statutory health and social care quality regulator), place some emphasis on nutrition, the dining environment and the mealtime experience. However, comparatively little attention is given to nutritional provision and its monitoring (particularly for TMDs), or to training for catering staff in TMD preparation. Ireland needs comprehensive national interdisciplinary LTC menu planning guidance. Such guidance should include nutrition standards for both nutritionally well and nutritionally vulnerable residents, promote the use of food-based enrichment strategies and fortified food products, and support training for long-term care staff, including catering departments. This training should emphasise the risk of malnutrition among residents consuming TMDs and the importance of preparing texture-modified meals that meet recommended nutritional targets. In the absence of national LTC-specific guidance, this research can serve as an early empirical signal to inform the forthcoming Food and Nutrition Policy for Older Persons Services and updated national statutory standards.

The current research highlights important gaps in the nutritional provision of TMDs. These findings support the need for a structured research strategy in this area. Initial studies should examine provision of TMDs across multiple LTC sites and over complete menu cycles to improve generalisability and identify inter-facility variation and modifiable practices. Subsequent research should investigate the relationship between TMD provision and consumption, through weighed intake (plate-waste) methods, while also examining the role of ONS and food-based enrichment. Further work linking provision and intake to indicators of nutritional status, such as weight change, body mass index, handgrip strength or other sarcopenia indices, micronutrient status, is also required. Ultimately, prospective studies should determine whether improved TMD planning and food-based enrichment strategies lead to clinically meaningful outcomes in LTC, including reduced falls, infections, pressure ulcers, hospitalisations and mortality.

### 4.4 Strengths and limitations

This study is the first known evaluation of TMD provision in an Irish LTC facility, providing novel insights into an under-researched area. The interdisciplinary research team, comprising nutrition and dietetics, speech and language therapy and population health, strengthened the study design, interpretation and contextualisation of findings. Data collection was conducted using a hierarchical, standardised quantification approach to minimise measurement error. Detailed collaboration with catering staff enabled accurate reconstruction of recipes, strengthening the validity of the nutrient analysis. Nutrient analysis was undertaken using validated software.

Several limitations should be noted. The analysis was based on two representative menu days, rather than a full cycle, which may not capture the full variability of provision. The study examined menu provision, rather than actual intake however, menu quality represents a crucial determinant of dietary intake. Vitamin B6, a nutrient of concern for older adults, was not analysed, representing an oversight, however it does not affect the core findings related to key nutrients of concern. As a single-site study, the generalisability of the specific numeric findings is limited and should be interpreted as illustrative of patterns that require confirmation across multiple facilities. However, the use of internationally recognised nutrition standards and transparent, replicable methods supports the methodological transferability of the approach to other LTC facilities. Future research incorporating multiple sites and full menu cycles would strengthen external validity of findings on TMD provision and further inform national guidance for TMD provision in Irish LTC settings.

## 5 Conclusions

This study evaluated the nutritional provision of TMDs in a LTC facility for older adults in Ireland, against established UK and Canadian menu standards and relevant reference values. Across most TMD Levels, mean daily and per-meal energy and protein did not meet recommended targets. Although daily sodium targets were met, all levels were high in total and saturated fat and consistently low in dietary fibre. Provision of several micronutrients was below dietary reference values, including vitamin D (except Level 3) vitamin C, iron, magnesium, potassium and zinc, consistent with previous findings from the literature that TMDs may provide lower nutrient levels, compared to regular diets. In contrast, the high calcium content of Level 3 diets, driven by extensive use of whole milk as a dilutant, may pose a risk of excessive intake for residents also consuming calcium supplements.

Overall, these findings demonstrate systematic imbalances in nutrient provision within the evaluated LTC menu and suggest potential gaps in menu planning and food-based enrichment practices at facility level. Addressing these issues may require the development of national food and hydration guidance for older persons’ services, alongside targeted training for catering staff to support the provision of safe TMDs that meet recommended nutritional targets.

This work also identifies priority areas for future research. Future work should evaluate TMD provision across multiple LTC sites and menu cycles, examine the relationship between provision and consumption, and determine whether improvements in TMD planning and enrichment strategies translate into meaningful clinical outcomes for residents.

## 6 Glossary

**AI (Adequate Intake):** Recommended intake when evidence is insufficient to establish another dietary reference value. The AI is the average daily nutrient intake assumed to meet the needs of a healthy population.

**Dietary Reference Values (DRVs):** Umbrella term for a set of population-level nutrient reference values (e.g., Average requirement (AR) Population Reference Intake (PRI), Adequate Intake (AI), Reference Intake range for macronutrients (RI), Tolerable Upper Intake Level (UL)) used to guide population-level assessment, policy and dietary guidance.

**Food Enrichment:** Adding nutrient-dense ingredients (e.g., skimmed milk powder, cheese, nut butters) to increase energy or protein content of foods and beverages.

**Food Fortification:** The addition of micronutrients to food/drinks to increase its nutritional content (e.g., Avonmore Supermilk)

**IDDSI (International Dysphagia Diet Standardisation Initiative):** A global framework providing standardised levels and testing methods for food and drink textures.

**LTC (Long-Term Care):** Residential or nursing care facilities providing accommodation with care for older adults.

**Malnutrition:** Or Protein-Energy Malnutrition or Undernutrition: a state of energy and protein deficiency which is associated with functional impairment and a worse outcome from illness as well as being specifically reversible by nutritional support

**Nutritional Standards/Menu Standards:** Benchmark nutrient targets (e.g., BDA Nutrition & Hydration Digest, BDA Care Home Digest, Canadian Menu Planning in Long-Term Care guidance) used to evaluate nutritional provision in menusfor the older adult in LTC population in this study.

**Nutrient Provision:** The amount of a nutrient supplied by meals or menus, not necessarily consumed.

**ONS (Oral Nutritional Supplements):** Commercially prepared, nutrient-dense liquids or powders used to augment dietary intake.

**PRI (Population Reference Intake):** The intake level sufficient for nearly all individuals in a population group.

**Texture Levels (IDDSI Levels 3–6):** Specific gradations of food texture: Level 3: Liquidised; Level 4: Puréed; Level 5: Minced & Moist; Level 6: Soft & Bite-Sized

**TMD (Texture-Modified Diet):** Diets altered in texture (e.g., puréed, minced, soft) to support safe swallowing for individuals with dysphagia.

## Funding

This research did not receive any specific grant from funding agencies in the public, commercial, or not-for-profit sectors.

## Data statement

Data described in the manuscript are publicly and freely available without restriction on Figshare at https://doi.org/10.6084/m9.figshare.32051790.

## Declaration of generative AI and AI-assisted technologies in the manuscript preparation process

Nothing to declare

## CRediT authorship contribution statement

**Erica Deasy:** Writing – review & editing, Writing – original draft, Methodology, Investigation, Formal analysis, Data curation. **Hannah Sheedy:** Writing – review & editing, Writing – original draft, Methodology, Investigation, Formal analysis, Data curation. **Cathal O’ Hara:** Writing – review & editing, Validation, Software, Formal analysis, Data curation. **Niamh Walsh:** Writing – review & editing, Visualization, Validation, Investigation, Data curation, Conceptualization. **Pauline Dunne:** Writing – review & editing, Writing – original draft, Validation, Formal analysis, Conceptualization. **Maria McKenna:** Writing – review & editing, Writing – original draft, Validation, Formal analysis, Conceptualization. **Marie Hannon:** Writing – review & editing, Writing – original draft, Visualization, Conceptualization. **Maria Gleeson-Cary:** Writing – review & editing, Writing – original draft, Visualization, Conceptualization. **Gráinne Kent:** Writing – review & editing, Writing – original draft, Visualization, Validation, Supervision, Resources, Project administration, Methodology, Formal analysis, Data curation, Conceptualization.

## Declaration of competing interest

The authors declare that they have no known competing financial interests or personal relationships that could have appeared to influence the work reported in this paper.

## Figures and Tables

**Table:** 

Nutrient	Recommendation	UL
**Energy (kcal)**	1840–2772 kcal/d [18] 2000k cal/d [19] >2000 kcal/d [20] Breakfast:545 kcal [18] Lunch: 800 kcal [18] Evening meal: 800 kcal [18] Snacks & drinks: 564 kcal [18]	-
**Protein (g)**	79–92 g/d [18] 75 g/d [19] 100 g/d [20] Breakfast: 18 g [18], 20 g [19], 20–25 g [20] Lunch: 27 g [18], 30 g [19], 20–25 g [20] Evening meal: 27 g [18], 25 g [19], 20–25 g [20] Snacks & drinks: 20 g [18], 25 g [20]	-
**Total fat (g)**	30–35% energy [20]	-
**Saturated fat (g)**	<10% energy [27]	-
**Carbohydrate (g)**	45–60% energy [28]	-
**Free sugars (g)**	<5% [28], <10% [58] energy	-
**Dietary fibre (g)**	30 g/d [20]	-
**Riboflavin (mg)**	1.6 mg**^a^** [30]	-
**Total folate (µg)**	330 µg**^a^** DFE/d [30]	1000 µg/d^d^ [29]
**Vitamin B12 (µg)**	4 µg/d^b^ [30]	-
**Vitamin C (mg)**	Females: 95 mg/d**^a^** Males: 110 mg/ d**^a^** [30]	2000 mg/d [31]
**Vitamin D (µg)**	20 µg/d [15]	100 µg/d [29]
**Vitamin E (mg)**	Females: 11 mg/d^b^ Males: 13 mg/d^b^ [30]	300 mg/d [29]
**Vitamin K (µg)**	70 µg/d (Vitamin K1)^b^ [30]	-
**Sodium (mg)**	<3500 mg [20]	-
**Potassium (mg)**	3500 mg/d^b^ [30]	-
**Calcium (mg)**	950 mg/d**^a^** [30]	2500 mg/d [29]
**Iron (mg)**	11 mg/d**^a^** [30]	-
**Magnesium (mg)**	Females:300 mg/d**^b^** Males: 350 mg/d**^b^** [30]	250 mg/d^e^ [29]
**Zinc (mg)**	Females: 7.5–12.7 mg/d**^a^^^c^^** Males: 9.4–16.3 mg/d**^a^^^c^^** [30]	25 mg/d [29]

## Appendix 1 Relevant nutrient reference values and sources

UL= The Tolerable Upper Intake Level (UL); the maximum level of total chronic daily intake of a nutrient (from all dietary sources) which is not expected to pose a risk of adverse health effects to humans. DFE = Dietary folate equivalents (DFE); calculated from food folate and folic acid [µg DFE = µg food folate + (1.7 x µg folic acid)], accounting for the higher bioavailability of folic acid (e.g., from fortified foods).**^a^**Population Reference Intake (PRI) is the level of nutrient intake that is adequate for virtually all people in a population group. ^b^The Adequate Intake (AI) is used when there is not enough data to calculate an average requirement. The AI is the average observed nutrient level consumed daily by a typical healthy population that is assumed to be adequate for the population’s needs. ^c^dependent on level of phytate intake ^d^from folic acid only ^e^from supplements only - = No UL set.
